# Antinociceptive and Antioxidant Activities of Phytol *In Vivo* and *In Vitro* Models

**DOI:** 10.1155/2013/949452

**Published:** 2013-06-11

**Authors:** Camila Carolina de Menezes Patrício Santos, Mirian Stiebbe Salvadori, Vanine Gomes Mota, Luciana Muratori Costa, Antonia Amanda Cardoso de Almeida, Guilherme Antônio Lopes de Oliveira, Jéssica Pereira Costa, Damião Pergentino de Sousa, Rivelilson Mendes de Freitas, Reinaldo Nóbrega de Almeida

**Affiliations:** ^1^Unity Academy of Hearth, Federal University of Campina Grande (UFCG), 58109-900 Cuité, PB, Brazil; ^2^Health Sciences Center, Federal University of Paraíba (UFPB), 58051-900 João Pessoa, PB, Brazil; ^3^Laboratory of Research in Experimental Neurochemistry of Post-Graduation Program in Pharmaceutics Science, Federal University of Piauí (UFPI), 64.049-550 Teresina, PB, Brazil; ^4^Department of Physiology, Federal University Paraíba (UFPB), 58051-900 João Pessoa, PI, Brazil

## Abstract

The objective of the present study was to evaluate the antinociceptive effects of phytol using chemical and thermal models of nociception in mice and to assess its antioxidant effects *in vitro*. Phytol was administered intraperitoneally (i.p.) to mice at doses of 25, 50, 100, and 200 mg/kg. In the acetic acid-induced writhing test, phytol significantly reduced the number of contortions compared to the control group (*P* < 0.001). In the formalin test, phytol reduced significantly the amount of time spent in paw licking in both phases (the neurogenic and inflammatory phases), this effect being more pronounced in the second phase (*P* < 0.001). Phytol also provoked a significant increase in latency in the hot plate test. These antinociceptive effects did not impaire the motor performance, as shown in the rotarod test. Phytol demonstrated a strong antioxidant effect *in vitro* in its capacity to remove hydroxyl radicals and nitric oxide as well as to prevent the formation of thiobarbituric acid reactive substances (TBARS). Taken as a whole, these results show the pronounced antinociceptive effects of phytol in the nociception models used, both through its central and peripheral actions, but also its antioxidant properties demonstrated in the *in vitro* methods used.

## 1. Introduction

The sensation of pain accompanies the majority of human diseases, alerting the body to the presence of harmful stimuli [1]. Pain may be modulated by a series of behavioral events, since, in addition to transmission of the stimulus that is causing the pain, the process also involves different emotional, environmental, and cognitive factors [2, 3].

Nociception involves activating sensorial neurons that transmit the nociceptive stimulus at spinal and supraspinal levels [4, 5]. Furthermore, following tissue damage, nociceptors are activated through the release of various mediators such as excitatory amino acids, protons, peptides, and cytokines that, in turn, act on specific receptors, activating various signaling cascades, which will result in the nociceptor membrane depolarization through the activation of second messengers or the sodium or calcium entry into the cell [3, 6–8]. Substances that are able to block these signal pathways, both at central and peripheral levels, represent important tools for pain control [9].

The current pharmacological treatment of pain consists in three main groups: central analgesics (opioids), peripheral analgesics (nonsteroidal antiinflammatory drugs-NSAIDs), and adjuvant drugs (antidepressants, anticonvulsants, and local anesthetics) [10, 11]. The development of new drugs for this purpose is an important field of study.

Reactive oxygen species (ROS) and reactive nitrogen species (RNS) are involved in various physiological and pathological processes, including inflammation and pain [12]. ROS/RNS production and their strong chemical reactivity with biomolecules such as proteins, lipids, and DNA may result in some harmful alterations such as the destruction of cell membranes by lipid peroxidation and DNA denaturation, leading to changes in protein synthesis and cell duplication [13].

Medicinal plants contain a variety of bioactive compounds such as terpenes, which are composed of essential oils [14–16]. The terpenoids found in the greatest abundance in essential oils are the monoterpenes and the sesquiterpenes; however, diterpenes may be present when the essential oils are extracted using organic solvents [16].

Various studies have shown a possible association between the antinociceptive and antioxidant properties of a substance. In this respect, various compounds of vegetable origin found in abundance in nature have been evaluated. The terpenes with confirmed antinociceptive and antioxidant properties include the monoterpenes carvacrol [17] and citronellal [18], as well as the diterpene and rographolide [19].

**Figure 1:**
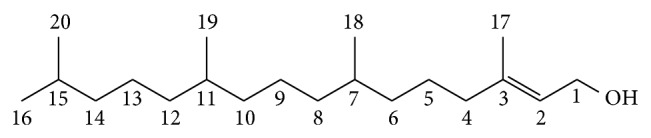
Chemical structure of phytol.

Phytol (3,7,11,15-tetramethylhexadec-2-en-1-ol) is a diterpene, a member of the group of branched-chain unsaturated alcohols (Figure 1) [20, 21]. It is the product of chlorophyll metabolism in plants; hence, phytol is abundantly available in nature. Phytol is known to inhibit the growth of *Staphylococcus aureus* [22] and to block the teratogenic effects of retinol [23]. Nevertheless, no reports have been published describing the effect of phytol on the central nervous system (CNS).

Therefore, the objective of the present study was to characterize the antinociceptive activity of phytol using *in vivo* animal models and to evaluate its antioxidant activity *in vitro*.

## 2. Experimental Procedures

### 2.1. Animals

Male Swiss albino mice weighing 25 to 35 grams were obtained from the Prof. Dr. Thomas George Laboratory of the Federal University of Paraiba and were subsequently separated into groups of 8 animals. The animals were kept under standard environmental temperature conditions (21 ± 1°C) with 12-hour light/dark cycles (lights on at 6:00 am) and with access to food and water *ad libitum* until 1 hour prior to the experimental procedures. The experiments were conducted between 12:00 and 17:00 pm. All the experimental protocols were approved by the Committee for the Care and Use of Animals of the Federal University of Paraiba (approval number 0104/11) and were conducted in accordance with the current guidelines for the care of laboratory animals and with the ethical guidelines for studies on experimental pain in conscious animals. The number of animals and the intensity of the noxious stimuli used were the minimum required to demonstrate the consistent effects of treatment with the drugs under evaluation.

### 2.2. Drugs

The following drugs were used: 2-deoxyribose, acetic acid, AAPH (2,2′-azobis[2-methylpropionamidine]dihydrochloride), diazepam, formalin, Griess reagent, hydrogen peroxide, indomethacin, morphine, phosphate buffer, sodium nitroprusside, thiobarbituric acid (TBA), thiobarbituric acid reactive species (TBARS), trichloroacetic acid, Trolox (6-hydroxy-2,5,7,8-tetramethylchroman-2-carboxylic acid), and phytol (97% pure) (Figure 1). All were purchased from Aldrich (USA).

### 2.3. *In Vivo* Experiments: Antinociceptive Activity

#### 2.3.1. Acetic Acid-Induced Writhing Test

This test was conducted according to the description published by Koster et al. [24]. The presence of abdominal contortions induced by an intraperitoneal injection of acetic acid was considered nociceptive behavior, characterized by abdominal muscle contractions followed by stretching of the hind limbs [25, 26]. For this experiment, groups of 8 mice received the following types of pretreatment: vehicle, phytol at the doses of 25, 50, 100, and 200 mg/kg, indomethacin (10 mg/kg), or morphine (10 mg/kg). Thirty minutes after pretreatment, the animals received intraperitoneal injections of a 0.85% solution of acetic acid (0.1 mL/10 g) and were then placed in individual polyethylene boxes. Five minutes later, the number of abdominal contortions was recorded during a 10-minute observation period [27].

A significant reduction in the number of contortions compared to the group treated with vehicle alone was considered to constitute an antinociceptive response [28–30].

#### 2.3.2. Formalin Test

The formalin test was conducted in a similar manner to that described by Hunskaar and Hole [31]. In this test, a formalin solution is injected into the subplantar region of the animal's paw, provoking stimulation of nociceptors [32]. The nociceptive response consists of two phases, the first normally occurring in the first five minutes following the formalin injection (neurogenic response) and the second phase occurring 15–30 minutes after the formalin injection (inflammatory response). Groups of 8 mice were used in this experiment and received the following types of pretreatment: vehicle (control group), phytol (25, 50, 100, and 200 mg/kg), morphine (10 mg/kg), or indomethacin (10 mg/kg). Thirty minutes later, 20 *μ*L of a 2.5% formalin solution (0.92% formaldehyde in saline solution) was injected into the subplantar region of the right hind paw of each mouse. These animals were then placed in the observation boxes, and the total time that the animals spent licking the paw in which the formalin injection was given was recorded during the first five minutes (first phase) and from 15 minutes to 30 minutes following the injection (second phase). Antinociception was defined as a reduction in paw-licking time compared to the control group [26, 33].

#### 2.3.3. Hot Plate Test

This method, first described by Woolfe and Macdonald [34], consists of quantifying the reaction time to the thermal stimulus caused by a plate heated to a temperature of 53 ± 1°C, based on a pain threshold that, depending on the type of fiber, ranges from 43 to 53°C [3]. Nociception is classified as present when the animal raises (an attempt to jump) or licks one of its hind paws [10, 33]. All the animals were submitted to initial screening 24 hours prior to the experiment. The animals that spent more than 15 seconds on the hot plate (model 7406, LE, Letica Scientific Instruments, Panlab S.L., Spain) without reacting to the thermal stimulus were eliminated from the experiment. The mice were divided into groups of 8 animals each and treated with vehicle (0.1 mL/10 g), phytol at the doses of 25, 50, 100, and 200 mg/kg, and morphine (10 mg/kg) intraperitoneally. The evaluations were carried out at intervals of 30, 60, and 120 minutes after the respective treatments. The animals were left on the plate for a maximum of 30 seconds to avoid tissue lesions [10, 35].

### 2.4. Evaluation of Locomotor Activity

This procedure was first described by Dunham and Miya [36] and is used to detect motor incoordination caused by pharmacological agents such as muscle relaxants or drugs that depress the central nervous system [37]. The rotarod device consists of a rod of 35 cm in length and 54 cm in width that rotates at an adjustable speed at a height of 45 cm. It is separated into four equal segments by plastic discs (Insight, model EFF 412). The animals were first screened without the administration of any substances, the selection criterion being to remain on the rotating rod of the device at a constant speed of 7 rotations per minute (7 rpm) for at least three minutes [38, 39]. Twenty-four hours after screening, the selected mice were divided into groups of 8 animals each. The total time during which they remained on the rod was evaluated, in up to three repetitions, for three minutes, at different time intervals (30, 60, and 120 minutes) following an intraperitoneal injection of phytol (25, 50, 100, and 200 mg/kg), diazepam (4 mg/kg), or vehicle [40].

### 2.5. *In Vitro* Experiments: Antioxidant Activity

#### 2.5.1. Thiobarbituric Acid Reactive Species (TBARS) Assay

A TBARS assay was used to quantify lipid peroxidation *in vitro* [41], and an adapted TBARS method was used to measure the antioxidant capacity of phytol *in vitro* using egg yolk homogenates as a lipid-rich substrate [17]. Briefly, egg yolk was homogenized (1% w/v) in 20 mM phosphate buffer (pH 7.4), and 1 mL of homogenate was sonicated and then homogenized with 0.1 mL of phytol at different concentrations. Lipid peroxidation was induced adding 0.1 mL of AAPH solution (0.12 M). The control group received only the vehicle (0.05% Tween 80 dissolved in 0.9% saline solution). Reactions were allowed to occur for 30 minutes at 37°C. After cooling, samples (0.5 mL) were centrifuged with 0.5 mL of trichloroacetic acid (15%) at 1200 g for 10 minutes. An aliquot of 0.5 mL of the supernatant was mixed with 0.5 mL thiobarbituric acid (TBA) (0.67%) and heated at 95°C for 30 minutes. After cooling, absorbance of the samples was measured on a spectrophotometer at 532 nm. The results were expressed as the percentage of TBARS formed by AAPH alone (induced control).

#### 2.5.2. Hydroxyl Radical Scavenging Activity

The formation of ^*∙*^OH (hydroxyl radical) by Fenton's reaction was quantified using 2-deoxyribose oxidative degradation [42]. The principle of the assay is quantification of the 2-deoxyribose degradation product, malondialdehyde, through its condensation with 2-thiobarbituric acid (TBA). Briefly, typical reactions were triggered by adding Fe^2+^ (final concentration of 6 mM FeSO_4_) to solutions containing 5 mM 2-deoxyribose, 100 mM H_2_O_2_, and 20 mM phosphate buffer (pH 7.2). To measure the antioxidant activity of phytol against hydroxyl radicals, different concentrations of phytol were added to the system before the addition of Fe^2+^. Reactions were carried out for 15 minutes at room temperature and were stopped by adding 4% phosphoric acid (v/v) followed by 1% TBA (w/v, in 50 mM NaOH). Solutions were boiled for 15 minutes at 95°C and then cooled at room temperature. Absorbance was measured at 532 nm, and results were expressed as MDA equivalents formed by Fe^2+^ and H_2_O_2_.

#### 2.5.3. Scavenging Activity of Nitric Oxide (NO)

Nitric oxide was generated from the spontaneous decomposition of sodium nitroprusside in 20 mM phosphate buffer (pH 7.4). Once generated, NO interacts with oxygen to produce nitrite ions, which were measured using the Griess reaction [43]. The reaction mixture (1 mL) containing 10 mM sodium nitroprusside (SNP) in phosphate buffer and phytol at different concentrations was incubated at 37°C for 1 hour. A 0.5 mL aliquot was taken and homogenized with 0.5 mL Griess reagent. The absorbance of the chromophore formed was measured at 540 nm. The extent to which the nitric oxide generated was inhibited was measured by comparing the absorbance values of negative controls (only 10 mM sodium nitroprusside and vehicle) and assay preparations. Results were expressed as percentages of nitrite formed by SNP alone.

## 3. Statistical Analysis

The data obtained are reported as means ± SEM and were evaluated using one-way analysis of variance followed by Dunnett's test or Student's *t*-test followed by the Neuman-Keuls test. Differences were considered to be statistically significant when *P* values were <0.05. Data were analyzed using the GraphPad Prism software version 5.01 (1992–2007, GraphPad Software Inc.). The percentage of inhibition by an antinociceptive agent was determined using the following formula [44]:

(1) $$\begin{array}{c} \%\text{Inhibition}=\left[\frac{\left(\text{control}-\text{experiment}\right)}{\text{control}}\right]\times 100. \end{array}$$

## 4. Results

### 4.1. Effect of Phytol on Acetic Acid-Induced Writhing

**Figure 2:**
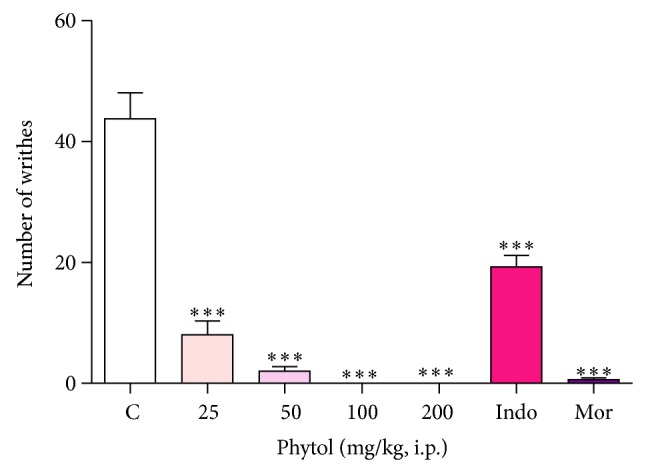
Effect of phytol (25, 50, 100, and 200 mg/kg, i.p.), indomethacin (10 mg/kg, i.p.), and morphine (10 mg/kg, i.p.) against acetic acid-induced abdominal writhing test in mice. Each column represents the mean ± SEM of 8 mice. Control values indicate the animals injected with saline + Tween 80. ∗∗∗*P* < 0.001 compared with the control group (one-way ANOVA followed by Dunnett's test).

Phytol (25, 50, 100, and 200 mg/kg, i.p.) significantly reduced the number of abdominal contortions induced by acetic acid in mice compared to the control group, with 81.7%, 95.4%, 100%, and 100% of inhibition, respectively, (*P* < 0.001) (Figure 2). Morphine (10 mg/kg, i.p.) and indomethacin (10 mg/kg, i.p.), used as standards, also inhibited this parameter, with 98.6% and 56.0% of inhibition, respectively.

### 4.2. Effect of Phytol on Formalin-Induced Nociception

**Figure 3:**
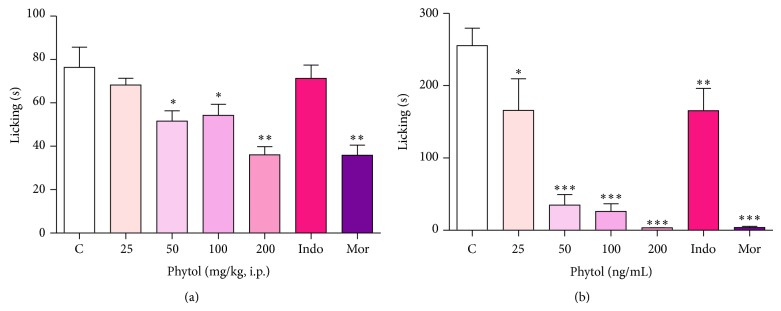
Effect of phytol (25, 50, 100, and 200 mg/kg, i.p.), indomethacin (10 mg/kg, i.p.), and morphine (10 mg/kg, i.p.) against formalin-induced paw licking test in mice on the first phase (a) and second phase (b). Each column represents the mean ± SEM of 8 mice. Control values indicate the animals injected with saline + Tween 80. ∗*P* < 0.05; ∗∗*P* < 0.01; ∗∗∗*P* < 0.001 compared with the control group (one-way ANOVA followed by Dunnett's test).

Treatment with phytol (25, 50, 100, and 200 mg/kg, i.p.) led to a significant reduction in both phases of the formalin test: the neurogenic phase (0–5 minutes, Figure 3(a)) and the inflammatory phase (15–30 minutes, Figure 3(b)). 

The effect of phytol was better in the inflammatory phase than in the neurogenic phase at all doses but particularly at the 200-mg/kg dose in which 98.7% of inhibition was achieved. Morphine (10 mg/kg, i.p.) provoked a marked inhibition (*P* < 0.001) in both phases: 53.0% in the neurogenic phase and 98.5% in the inflammatory phase. In contrast, the effect of indomethacin (10 mg/kg, i.p.) was significant in the inflammatory phase (43.3%) but not in the neurogenic phase.

### 4.3. Effect of Phytol in the Hot Plate Test

Table 1 shows the effects of phytol (25, 50, 100, or 200 mg/kg, ip) in the hot plate. In the assessment conducted at 30 minutes following the administration of phytol, a significant increase occurred in latency in response to the thermal stimulus at all doses compared to the control group.

### 4.4. Effect of Phytol on Locomotor Activity

As shown in Table 2, phytol (25, 50, 100, and 200 mg/kg, i.p.) did not cause any significant change in the motor performance of mice submitted to the rotarod test compared to the control group. Nevertheless, diazepam (4 mg/kg, i.p.), used as the standard drug, significantly reduced the time the animals remained on the rotarod at 30 minutes following administration (83.5 ± 17.9) compared to the control group (178.3 ± 0.9).

### 4.5. Effect of Phytol on TBARS Production

**Figure 4:**
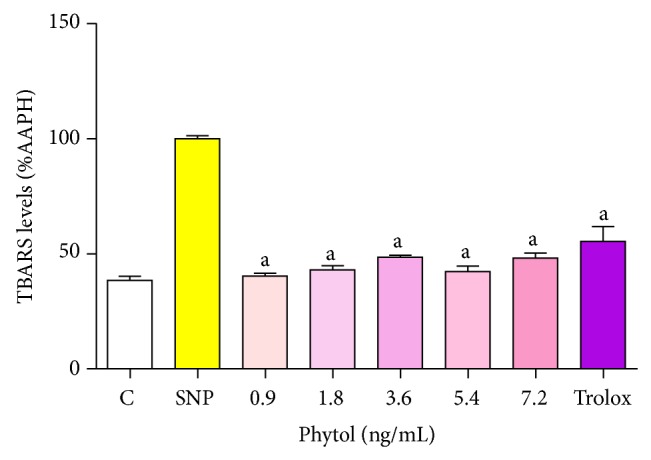
Effects of phytol on TBARS level *in vitro*. Lipid extracted from egg yolk was subjected to oxidative damage by incubation with AAPH, and the ability of different concentrations of phytol to prevent TBARS formation was analyzed. Control means basal lipid peroxidation with vehicle alone (0.05% Tween 80 dissolved in 0.9% saline); AAPH alone group is considered as 100% of oxidative damage. Values represent mean ± SEM (*n* = 8), experiments in duplicate. ^a^ *P* < 0.001 versus AAPH (ANOVA and *t*-Student-Neuman-Keuls tests).

Phytol (0.9, 1.8, 3.6, 5.4, and 7.2 ng/mL) resulted in a significant reduction in TBARS production at all concentrations tested, while Trolox led to a reduction of 48.12% in TBARS production (Figure 4). The 7.2 ng/mL concentration of phytol no produce an increase in the removal of TBARS levels when compared with 0.9, 1.8, 3.6, and 5.4 ng/mL concentrations, respectively (*P* > 0.05).

### 4.6. Effect of Phytol on Scavenging Activity of Hydroxyl Radicals

**Figure 5:**
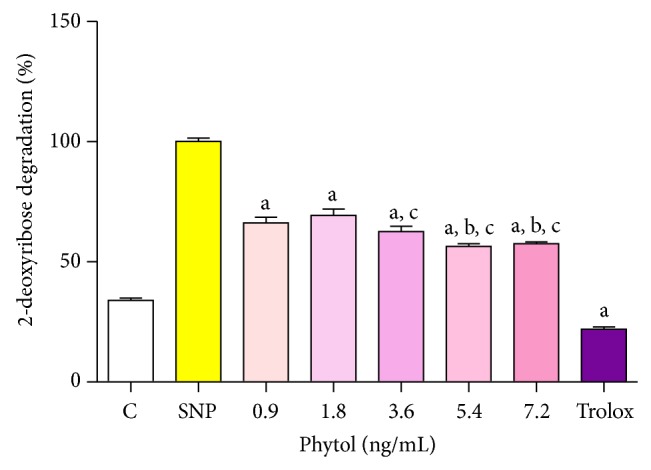
OH-scavenging activity of phytol. Hydroxyl radical scavenging activity was quantified using 2-deoxyribose oxidative degradation *in vitro*, which produces malondialdehyde by condensation with 2-thiobarbituric acid (TBA). System is MDA production from 2-deoxyribose degradation with FeSO_4_ and H_2_O_2_ alone. Other groups denote MDA production by FeSO_4_ and H_2_O_2_ in the presence of different concentrations of phytol (0.9, 1.8, 3.6, 5.4, and 7.2 ng/mL). Values represent mean ± SEM (*n* = 8), experiments in duplicate. ^a^ *P* < 0.001 versus system; ^b^ *P* < 0.001 versus 0.9 ng/mL; ^c^ *P* < 0.001 versus 1.8 ng/mL; (ANOVA and *t*-Student-Neuman-Keuls test).

As shown in Figure 5, phytol (0.9, 1.8, 3.6, 5.4, and 7.2 ng/mL) resulted in the removal of hydroxyl radicals at all the concentrations tested compared to the control group. The 0.9 ng/mL concentration of phytol led to an increase in the removal of hydroxyl radicals of 9.66% and 8.62% compared to the 5.4 and 7.2 ng/mL concentrations, respectively. The 1.8 ng/mL concentration of phytol resulted in an increase in the removal of the hydroxyl radicals of 6.69%, 12.84%, and 11.80% compared to the concentrations of 3.6, 5.4, and 7.2 ng/mL, respectively. Trolox resulted in the removal of 78.06% of the hydroxyl radicals.

### 4.7. Effect of Phytol on Scavenging Activity against Nitrite Production

**Figure 6:**
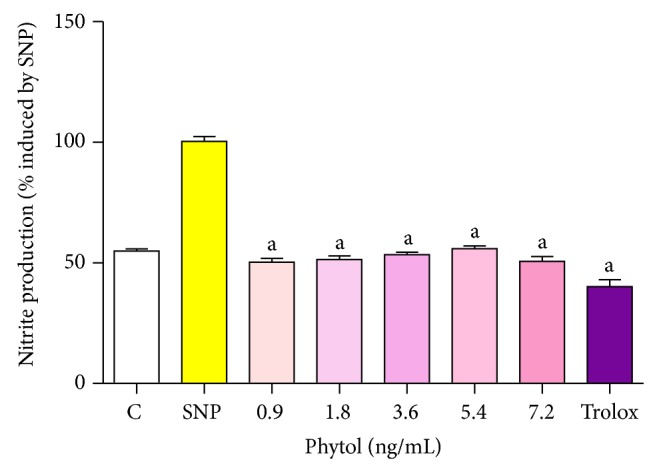
No scavenging activity of phytol. Control means basal NO production of vehicle (0.05% Tween 80 dissolved in 0.9% saline) in the absence of an NO generator source (without SNP); SNP group reflects nitrite production by sodium nitroprusside alone, considered 100% of NO production. The effect of different concentrations of phytol against SNP was determined by the Griess method. Values represent mean ± SEM (*n* = 8), experiments in duplicate. ^a^ *P* < 0.001 versus SNP (ANOVA and *t*-Student-Neuman-Keuls test).

Phytol (0.9, 1.8, 3.6, 5.4, and 7.2 ng/mL) was able to reduce nitrite production at all the concentrations evaluated, as shown in Figure 6. Trolox, a synthetic analog of *α*-tocopherol used as a standard antioxidant, reduced nitrite production by 59.76%. The 7.2 ng/mL concentration of phytol no led to an increase in the removal of nitrite production when compared to the 0.9, 1.8, 3.6, and 5.4 ng/mL concentrations, respectively, (*P* > 0.05).

## 5. Discussion

The results of this study showed that phytol has an antinociceptive effect in several models of nociception as well as antioxidant properties against free radicals generated *in vitro*.

In our study, we used chemical (acetic acid and formalin tests) and thermal (hot plate test) models of nociception in mice to investigate the antinociceptive effect of phytol [45]. The use of multiple models is essential for the detection of the antinociceptive properties of a substance, since stimuli different types of pain reveal the antinociceptive profile of the drug [46, 47].

The test of abdominal contortions induced by acetic acid is described as a classic model of inflammatory visceral nociception and is widely used as a pharmacological tool for the evaluation of new agents with analgesic and/or anti-inflammatory properties [28]. It is a simple and very sensitive method, since it is able to detect the antinociceptive effect of central (opioids) and peripheral (NSAIDs) analgesics, muscle relaxants, and sedatives [48–50].

At the cell level, the protons resulting from dissociation of acetic acid depolarize sensory neurons by interacting with acid-sensitive ion channels (ASICs) or indirectly by promoting of the release of several inflammatory mediators (prostaglandins, histamine, substance P, among others) [1, 3], stimulating primary afferent neurons to increase release of glutamate and aspartate [51]. In addition, the nociception caused by acetic acid is associated with increased production of lipoxygenase in the peritoneal cavity, which promotes the stimulation of primary sensory C fibers [49, 52, 53].

Our results showed that phytol produces an inhibition of nociception in mice by significantly reducing the number of abdominal contortions. The results also showed that the drugs used as standard, indomethacin and morphine, caused a significant inhibition of this parameter. Although these results may suggest a powerful antinociceptive effect of phytol, this test alone is unable to demonstrate whether the antinociception was central or peripheral.

The formalin test, which consists of a biphasic model of nociception, was used to assess the mechanism by which an animal responds to continuous nociception generated by tissue damage [50, 54].

The data obtained show that phytol suppressed both phases in the formalin test, and the effect being more pronounced in the second phase, which suggests that phytol has both central and peripheral antinociceptive activity and may be associated with an anti-inflammatory effect. Furthermore, we demonstrated that morphine inhibited both phases, whereas indomethacin inhibited only the second phase.

To provide a confirmation of the central antinociceptive effect of phytol, we used the hot plate test, since this test is sensitive and specific for drugs that act through a central mechanism [55–57], while peripherally acting analgesics are inactive [47, 58].

The results indicate that phytol in the tested doses increased reaction time (latency) to the thermal stimulus. Thus, the increase in nociceptive threshold of mice treated with phytol along with the reduction of nociception in the first phase of the formalin test revealed strong evidence of its antinociceptive activity mediated by central mechanisms.

In doses tested, there was no prejudice to the locomotor performance of animals. Thus, the possibility of sedative effect which causes loss of coordination is excluded.

The antioxidant activity of phytol was also investigated in this study, since there is a relationship between these two activities, as demonstrated by Guimarães et al. [17]. Several lines evidence indicate that oxidative stress has a crucial role in signaling of nociception and is involved in the process of pain (neuropathic and inflammatory) [59, 60].

From the *in vitro* methods used, we showed that phytol was able to reduce the production of free radicals, and this activity can be attributed to their structural feature, since phytol is a branched-chain unsaturated alcohol and its antioxidant properties may be related to the hydroxyl group (OH) present in its molecule. Probably, phytol, by reacting with a free radical, donates hydrogen atoms with an unpaired electron (H^*∙*^), converting free radicals into less reactive species [17, 61].

The antioxidant activity was evaluated by TBARS assay that is a method used to quantify lipid peroxidation, which corresponds to a cell membrane damage caused by oxidative stress. The AAPH was used as a generator of free radicals. Its decomposition produces molecular nitrogen and carbonyl radicals, which in turn react with thiobarbituric acid, resulting in the formation of TBARS [13, 62–64].

Phytol, at all concentrations tested, was able to prevent lipid peroxidation by inhibiting the amount of TBARS formed. Similar results were obtained with Trolox, a synthetic analogue of *α*-tocopherol, used as standard antioxidant. This result suggests that phytol can exert an antioxidant activity that protects the lipid biomolecules [64].

Scavenger assay is a method widely used to evaluate the antioxidant activity of a substance based on the ability scavenger of free radicals formed in less reactive species [65]. The ability of a substance scavenging OH^*∙*^ is directly related to its antioxidant activity. The OH^*∙*^ is an extremely reactive species capable of causing damage to DNA, proteins, and lipids [64, 66–68].

In our study, we demonstrated that phytol produced the removal of OH^*∙*^, exhibiting antioxidant activity which may be capable of inhibiting cell damage caused by this radical. Trolox (positive control) also significantly reduced the amount of this radical.

Another method used was the nitric oxide- (NO-) scavenging assay. This method is based on the production of NO from the decomposition of sodium nitroprusside in aqueous solution. NO, in turn, interacts with oxygen to produce nitrite ions, which have strong oxidizing power, reacting with various biological molecules, which leads to cell damage [17, 69]. Substances with acting NO-scavenger compete with oxygen, leading to reduced production of nitrite, characterizing the antioxidant activity [64, 70]. 

In this study, phytol significantly decreased the production of nitrite, demonstrating once again its antioxidant property against damage caused by free radicals. Several studies show that inhibition of reactive oxygen (ROS) and reactive nitrogen (RNS) species may be associated with control of the central and peripheral sensitization in various pain states [17, 71].

The results suggest that phytol at concentrations tested is most efficient in removal of the hydroxyl radical, and once produced, a great reduction on formation of this free radical increased significantly in function of concentration tested and when compared to antioxidants effects against the TBARS and nitrite levels.

## 6. Conclusions

Therefore, the results obtained in this study demonstrate that phytol produces antinociceptive activity in mice, suggesting central and peripheral effect, without changing the motor function of animals. The antinociceptive activity may be associated with the antioxidant activity of phytol as demonstrated by *in vitro* methods used. Therefore, the results obtained in this study demonstrate that phytol produces antinociceptive activity in mice, suggesting central and peripheral effect, without changing the motor function of animals. 

The antinociceptive activity may be associated with the antioxidant activity of phytol as demonstrated by *in vitro* methods used. More studies are needed to elucidate the possible action mechanisms that mediate the central and peripheral antinociception, as well as the antioxidant activity of phytol against other free radical generating systems and with other different concentrations of this diterpene evaluated in this study, since the concentrations tested were more efficient in removing the hydroxyl radical.

## Figures and Tables

**Table 1:** Effects of phytol (25, 50, 100, and 200 mg/kg, i.p.) and morphine (10 mg/kg, i.p.) on heat-induced nociception in mice (hot plate test).

Tratament (mg/kg, i.p.)	Latencies (s)		
	30 min	60 min	120 min
Control	3.8 ± 0.4	3.9 ± 0.4	3.7 ± 0.7
Phytol 25	9.6 ± 0.9∗∗	7.9 ± 1.2	6.2 ± 1.0
Phytol 50	11.0 ± 2.0∗∗∗	10.3 ± 1.9∗∗	8.8 ± 1.0∗∗
Phytol 100	10.1 ± 1.0∗∗	7.3 ± 0.7	8.0 ± 1.2∗
Phytol 200	9.3 ± 0.6∗	7.6 ± 1.6	7.3 ± 1.0
Morphine 10	15.0 ± 1.5∗∗∗	13.6 ± 1.4∗∗∗	7.2 ± 0.6

Latency values are expressed as mean ± SEM (*n* = 8). Control values indicate the animals injected with saline + Tween 80. ∗*P* < 0.05; ∗∗*P* < 0.01; ∗∗∗*P* < 0.001 compared with the control group (one-way ANOVA followed by Dunnett's test).

**Table 2:** Effect of phytol (25, 50, 100, and 200 mg/kg, i.p.) and diazepam (4 mg/kg, i.p.) on the rotarod test in mice 30 min, 60 min, and 120 min after treatment.

Tratament (mg/kg, i.p.)	Periods		
	30 min	60 min	120 min
Control	178.3 ± 0.9	178.1 ± 0.9	179.1 ± 0.4
Phytol 25	174.3 ± 3.6	155.1 ± 15.8	167.6 ± 12.4
Phytol 50	179.5 ± 0.5	180.0 ± 0.0	174.6 ± 5.4
Phytol 100	173.0 ± 4.5	153.3 ± 17.6	163.6 ± 16.4
Phytol 200	165.0 ± 7.1	179.3 ± 0.5	177.8 ± 1.2
Diazepam 4	83.5 ± 17.9∗∗∗	144.5 ± 17.1	164.0 ± 9.5

The values represent the mean ± SEM (*n* = 8). Control values indicate the animals injected with saline + Tween 80. ∗∗∗*P* < 0.001 compared with the control group (Student's *t-*test followed by unpaired test).
